# Multiseptate gallbladder: A case report and literature review

**DOI:** 10.1097/MD.0000000000027992

**Published:** 2021-12-10

**Authors:** Yu-Min Hsieh, Yuli Lily Hsieh, Nien-Lu Wang, Pao-Shu Wu, Shu-Chao Weng

**Affiliations:** a Department of Pediatric Gastroenterology, Hepatology and Nutrition, MacKay Children's Hospital, Taipei, Taiwan; b Department of Pediatric Surgery, MacKay Memorial Hospital, Taipei, Taiwan; c Department of Pathology, MacKay Memorial Hospital, Taipei, Taiwan; d Mackay Junior College of Medicine, Nursing, and Management, Taipei, Taiwan; e Interfaculty Initiative in Health Policy, Harvard University, Boston, MA, United States; f Center for Health Decision Science, Harvard T.H. Chan School of Public Health, Boston, MA, United States.

**Keywords:** biliary symptoms, multiseptate gallbladder, rare congenital anomaly

## Abstract

**Rationale:**

Multiseptate gallbladder (MSG) is a rare congenital gallbladder anomaly. Between 1963 and June 2021, only 56 cases were reported. There is currently no treatment guideline for pediatric or adult cases of MSG.

**Patient concerns:**

A 14-year-old woman visited our out-patient clinic in September 2020 for epigastric pain that last for 6 months. Honeycomb appearance of the gallbladder was noted under ultrasonography.

**Diagnosis:**

The patient was diagnosed with MSG. The diagnosis was confirmed through computed tomography and magnetic resonance cholangiopancreatography.

**Interventions:**

Cholecystectomy was performed.

**Outcomes:**

Epigastric pain showed limited improvement after the surgery. Since she was diagnosed with gastritis at the same time, a proton-pump inhibitor was prescribed. Epigastric pain was eventually resolved.

**Lessons:**

MSG cases can undergo cholecystectomy and show good recovery without complications. However, concomitant treatment may be required to resolve in the presence of other symptoms such as epigastric pain.

## 1 Introduction

The multiseptate gallbladder (MSG) is a rare gallbladder anomaly. Between 1963 and June 2021, only 56 cases were reported in the English literature. These published case reports and case series describe the clinical presentations of MSG, the features of the diagnostic workup, as well as treatment and prognosis of MSG. Simon and Tandon reported the first case of a 32-year-old woman with upper abdominal and back pain that lasted for 3 weeks, revealing a “honeycomb-like” appearance within the gallbladder under ultrasonography (USG).^[1]^ The first pediatric case was published 3 years later, in which a 15-year-old woman with MSG had recurrent abdominal pain.^[2]^

Congenital anomalies of the gallbladder can be categorized based on their size, shape, position, and number. MSG is a rare congenital anomaly with distinct shapes. Since no malignant cases have been reported to date, MSG is considered a benign disorder.^[3]^ However, patients with MSG can suffer from other biliary anomalies. There have been several postulations regarding the mechanisms that contribute to the formation of MSG.^[4–7]^ However, the exact etiology remains unclear, and there is no consensus on how MSG should be treated.

## 2 Case presentation

A 14-year-old previously healthy Asian female visited the outpatient department with a chief complaint of epigastric cramping pain that lasted for 6 months. The patient did not have fever or jaundice. At the abdominal examination, epigastric tenderness was noted. Results of whole blood count, erythrocyte sedimentation rate, C-reactive protein, and biochemical tests including transaminase, bilirubin, amylase, lactic dehydrogenase, and alkaline phosphatase levels were within normal ranges.

**Figure 1:**
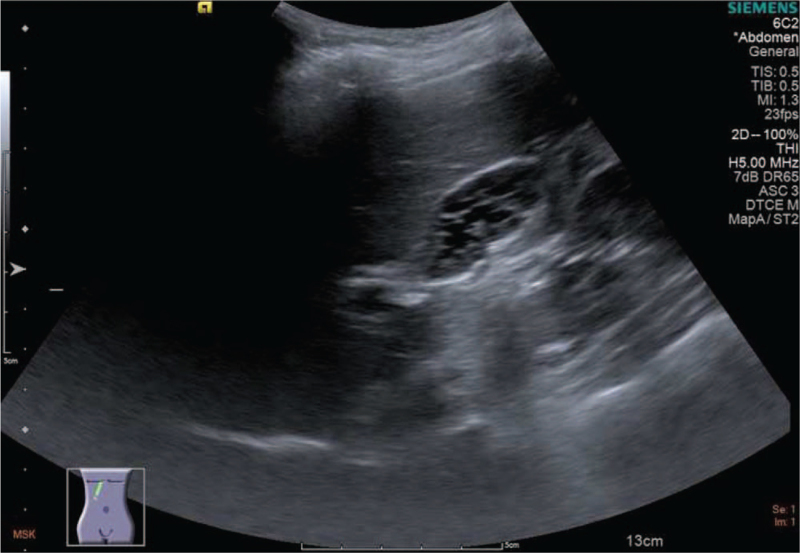
Abdominal echo: multiple thin septa in gallbladder without gallstone.

USG showed a multiple thin septa-bridged gallbladder with a honeycomb appearance, which is consistent with the clinical feature of a MSG (Fig. 1). The thickness of the gallbladder wall was normal, with no stones in the lumen. Neither pericholecystic fluid nor biliary dilatation was observed. No focal tenderness was observed in the gallbladder. To further examine the structure and rule out relevant anomalies, we arranged computed tomography and magnetic resonance cholangiopancreatography (MRCP). Computed tomography revealed a fine septum over the distal body of the gallbladder, some tiny polypoid hyperintensities along the inner wall of the gallbladder sac, and fluid-fluid level in the gallbladder. MRCP excluded intra- and extrahepatic biliary or pancreatic anomalies.

**Figure 2:**
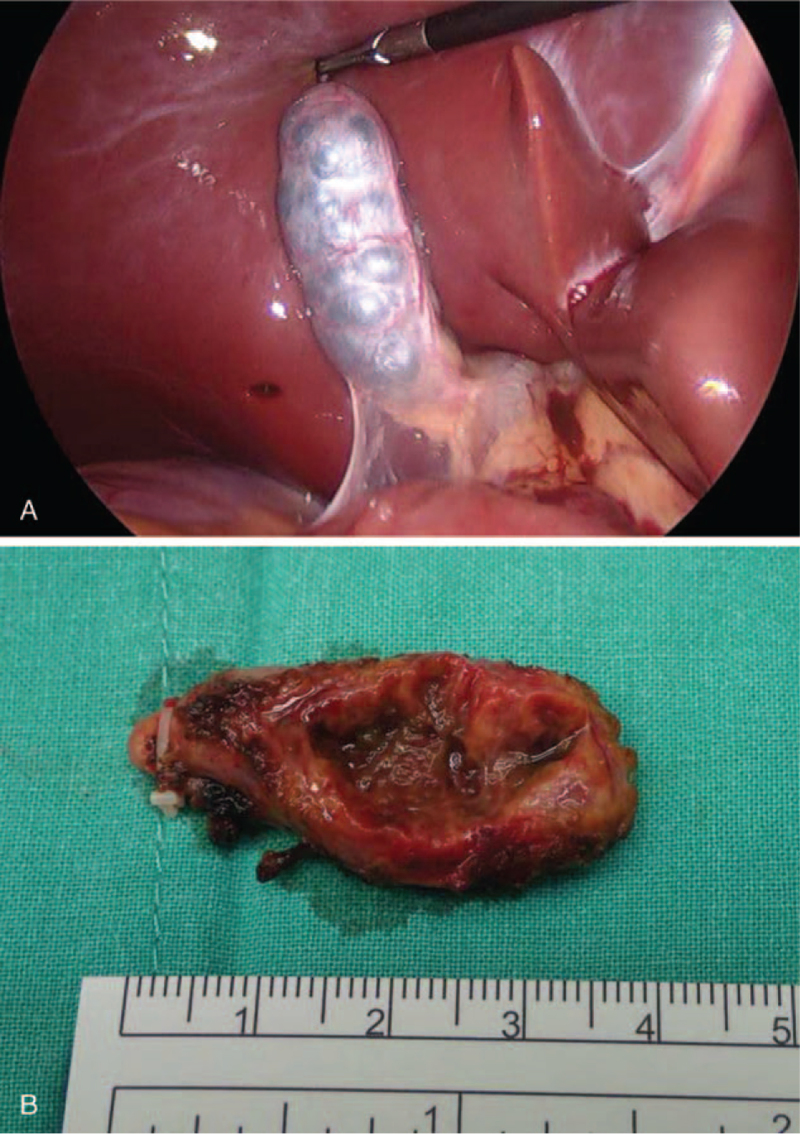
(A) Macroscopically, a grape-like appearance of the gallbladder can be visualized during the laparoscopic procedure. (B) The specimen consisted of a gallbladder measuring 3.5 × 2 × 1.5 cm in size.

**Figure 3:**
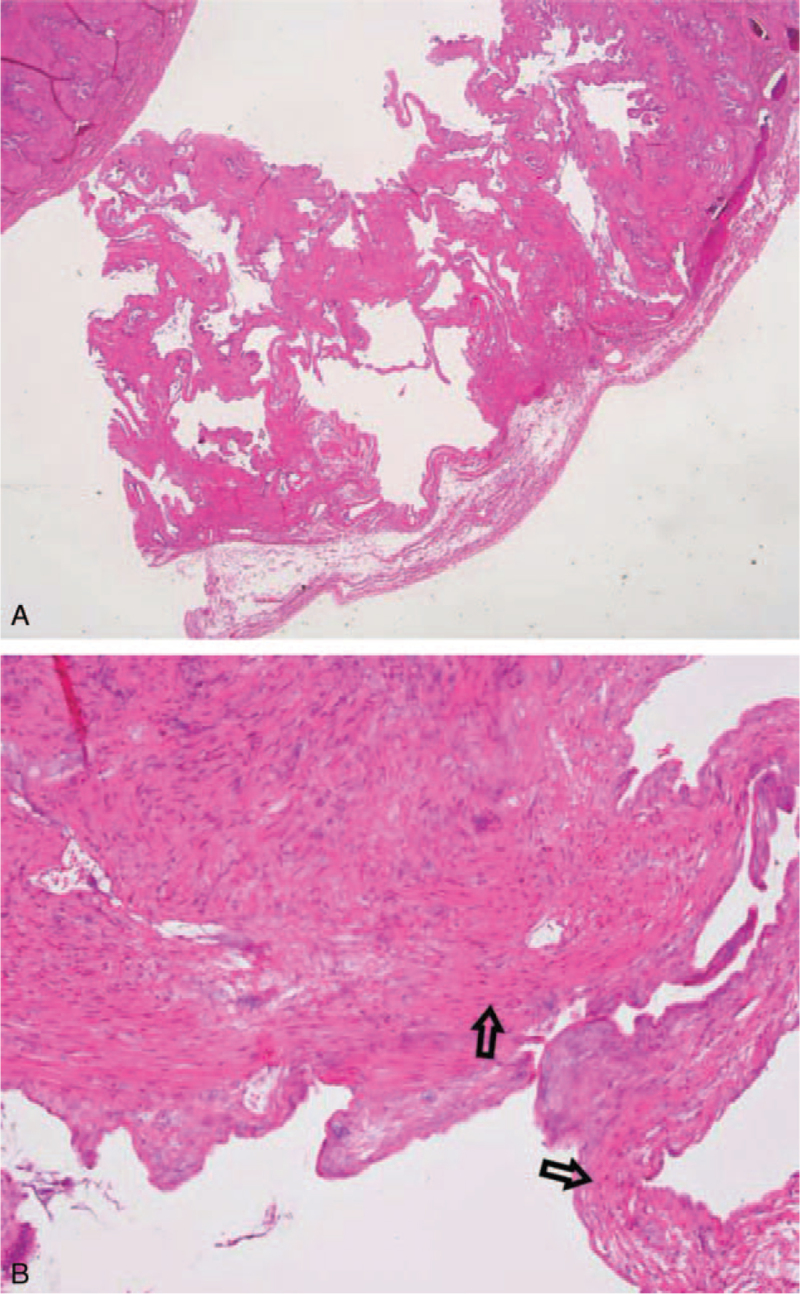
Pathology: (A) The serosa was smooth. On section, the wall was 0.5 cm in thickness. The mucosa appeared trabeculated, showing a picture compatible with the multiseptate gallbladder (H&E stain, 10×). (B) Examination under the oil immersion objective revealed muscle layer. Arrow: smooth muscle (H&E stain, 100×).

In the workup for epigastric pain, we performed an esophagogastroduodenoscopy. The patient was diagnosed with gastritis and gastric ulcers with no evidence of *Helicobacter* infection. She was treated with a proton pump inhibitor. Upon diagnosis of MSG, the patient chose to undergo laparoscopic cholecystectomy, even though the MSG can be left untreated and monitored through regular follow-up (Fig. 2A, B). The specimen was sent for pathology study. The histopathologic diagnosis revealed smooth serosa and trabeculated mucosa, with a muscle layer extending into the septa, indicating a multiseptate gallbladder (Fig. 3A, B). The surgery was uneventful, but her abdominal pain persisted after surgery. The epigastric pain eventually subsided as the patient continued to take a proton-pump inhibitor.

## 3 Discussion

To the best of our knowledge, this was the first case of MSG we cared for at our hospital. To better understand this rare anomaly, we conducted a literature review using the PubMed medical database with keywords “multiseptate gallbladder.” Only English literature was considered. Forty-two articles were included in this review. Data on the 57 cases in these 42 articles are summarized in Table 1.

**Table 1:** Demographic data of children and adults.

Children							
Year	Author	Age	Gender	Biliary symptoms	Associated anomaly	Treatment	Prognosis
1966	Haslam et al ^[2]^	15	F	Yes	Nil	Cholecystectomy	–^∗^
1985	Pery et al ^[15]^	8	M	Yes	Choledochal cyst	Choledochoduodenostomy	–
1990	Adear et al ^[26]^	12	F	No	Nil	Observation	NA^†^
1993	Strauss et al^[27]^	3	M	No	Nil	Not detailed	–
1993	Strauss et al^[21,27]^	9	F	Yes	Nil	Not detailed	–
1993	Strauss et al^[27]^	16	M	Yes	Nil	Not detailed	–
1993	Tan et al^[16]^	14	F	Yes	Choledochal cyst	Cholecystectomy and Hepatojejunostomy	Resolved
1996	Kong et al ^[23]^	9	M	Yes	Nil	Observation	Resolved
1998	Saddik^[28]^	10	M	No	Nil	Observation	NA
1999	Geremia et al^[10]^	10	M	Yes	biliary sludge	Observation	Resolved
2003	Kocako et al ^[25]^	9	M	Yes	Nil	Observation	Resolved
2004	Erdogmus et al ^[9]^	10	F	Yes	Cholelithiasis	Cholecystectomy	–
2004	Erdogmus et al^[9]^	12	M	No	Nil	Cholecystectomy	Resolved
2006	Bahadir et al ^[17]^	15d	M	Yes	Ectopic pancreas/ Choledochal cyst	Total excision of cyst with Roux-en-Y anastomosis	Resolved
2010	Demirpolat et al ^[29]^	5	F	No	Nil	Observation	Resolved
2011	Wanaguru et al ^[3]^	9m	F	No	Nil	Observation	NA
2011	Herliczek ^[30]^	11	M	No	Nil	Not detailed	Resolved
2019	La Mendola et al ^[11]^	3	F	Yes	Cholelithiasis	Cholecystectomy	Resolved
2020	Present case	14	F	Yes	Nil	Cholecystectomy	Not resolved
Adults							
1963	Simon et al^[1]^	32	F	Yes	Nil	Not detailed	–
1964	Bigg^[31]^	38	M	Yes	Nil	Cholecystectomy	Resolved
1968	Sachsse	50	M	Yes	Nil	Observation	–
1970	Bhagavan, et al ^[6]^	27	F	Yes	Hypoplasia of GB	Cholecystectomy	–
1973	Croce^[32]^	45	F	Yes	Cholelithiasis	Cholecystectomy	Resolved
1975	Arnaud et al	28	F	Yes	Cholelithiasis	Cholecystectomy	–
1975	Shaw et al ^[33]^	31	F	Yes	Nil	Cholecystectomy	Resolved
1975	Konishi et al	51	F	Yes	Nil	Cholecystectomy	–
1976	Bielby et al	57	F	Yes	Cholelithiasis	Cholecystectomy	–
1977	Jena et al ^[34]^	28	F	Yes	hypoplasia of GB	Cholecystectomy	–
1979	Okuda et al ^[35]^	37	M	Yes	Nil	Cholecystectomy	–
1981	Alawneh et al	44	F	Yes	Cholelithiasis	Cholecystectomy	–
1982	Toombs et al ^[36]^	22	F	Yes	Nil	Cholecystectomy	Resolved
1985	Oliva Oliva et al ^[37]^	24	F	Yes	Nil	Cholecystectomy	–
1987	Lev-Toaff et al^[24]^	23	F	No	Nil	Not detailed	–
1987	Lev-Toaff et al^[24]^	30	M	No	Nil	Observation	NA
1990	Isomoto et al ^[38]^	43	F	Yes	Nil	Cholecystectomy	–
1990	Vasinrapee et al ^[39]^	24	M	Yes	Not detailed	Not detailed	–
1994	Naritomi et al ^[40]^	45	F	Yes	Nil	Cholecystectomy	Resolved
1994	Hahm et al ^[41]^	49	F	Yes	Nil	Observation	Resolved
1996	Saimura et al ^[12]^	30	M	Yes	Nil	Cholecystectomy	Resolved
1997	Paciorek et al ^[42]^	25	F	No	Nil	Cholecystectomy	Resolved
2000	Miwa et al ^[43]^	70	F	No	Nil	Observation	NA
2002	Kapoor et al^[44]^	21	M	No	Nil	Observation	NA
2003	Erdogmus et al ^[9]^	23	M	No	Nil	Observation	–
2003	Erdogmus et al ^[9]^	33	F	No	Nil	Observation	–
2003	Erdogmus et al ^[9]^	40	F	No	Nil	Observation	–
2003	Erdogmus et al ^[9]^	45	M	Yes	Nil	Not detailed	–
2003	Erdogmus et al ^[9]^	40	F	Yes	Nil	Not detailed	–
2003	Erdogmus et al ^[45]^	20	F	Yes	Nil	Cholecystectomy	–
2004	Nakazawa et al^[14]^	56	F	Yes	Nil	Cholecystectomy	Resolved
2005	Yamamoto et al^[8]^	46	F	No	Anomalous pancreaticobiliary ductal union	Total gastrectomy/ Cholecystectomy	–
2006	Türkvatan et al^[18]^	62	M	Yes	Ectopic, hypoplastic gallbladder/ Choledochal cyst	Cholecystectomy/ Hepaticojejunostomy	–
2008	Yamasaki et al^[46]^	53	F	No	Nil	Gastrectomy/ Cholecystectomy	Resolved
2009	Rivera-Troche et al ^[7]^	19	F	No	Nil	Cholecystectomy	Resolved
2011	Karaca et al^[47]^	29	F	Yes	Nil	Cholecystectomy	Resolved
2017	Honrubia López et al ^[48]^	28	F	Yes	Nil	Observation	Resolved
2020	Singh et al^[4]^	49	F	Yes	Choledochal cyst	Not detailed	–

In this discussion, we defined choledochal cysts and anomalous arrangement of the pancreaticobiliary duct as pre-cancerous anomalies, given the risk of malignant progression. Biliary symptoms were defined as either right upper quadrant pain or epigastric pain, fever, nausea, vomiting, or jaundice. Individuals with “recurrent abdominal pain” and/or “abdominal pain” were sorted into group that did not have biliary symptoms.

### 3.1 Patient demographics

Out of the 57 cases, 19 cases (33%) were pediatric cases, with a gender ratio close to 1 (female:male = 9:10). The median age at diagnosis was 10 years (range: 15-day-old-16 years). Among the 38 adult cases (66%), the youngest case was 19 years old and the oldest was diagnosed at the age of 70 years. The median age at diagnosis was 35 years (Table 2A). Unlike pediatric cases, MSG is 2.8 times more prevalent in females than in their male counterparts.

**Table 2:** (A) Analysis of sex, median age at diagnosis, biliary symptoms, and associated anomalies in children and adults. (B) Analysis of biliary symptoms in children and adults^∗^.

(A)		
Variables	Children (n = 19)	Adult (n = 38)
Female, n (%)	9 (47.3)	28 (73.7)
Median age at diagnosis, *d* (range of age)	10 (15-day-old -16)	35 (19–70)
Biliary symptoms, n (%)	12 (63.1)	27 (71)
Anomalies, n (%)	6 (31.6)	9 (23.7)
Pre-cancerous anomalies, n (%)	3 (15.8)	3 (7.9)
Non-pre-cancerous anomalies, n (%)	3 (15.8)	6 (15.8)

(B)		
Variables	Children (n = 12)	Adult (n = 21)
Fever, n (%)	1 (8.3)	0
Jaundice, n (%)	3 (25)	0
Nausea/vomiting, n (%)	4 (33.3)	5 (23.8)
Right upper quadrant pain, n (%)	5 (41.7)	13 (61.9)
Epigastric pain, n (%)	4 (33.3)	7 (33.3)

### 3.2 Pathogenesis

There are several postulations to explain the formation of MSG. First, some suggested that MSG results from incomplete cavitation of the solid embryonic gallbladder because MSG cases do not have the muscle layer in the septa.^[1,5]^ Second, the “wrinkling theory” states that the gallbladder has a wrinkling appearance and creates invagination that fuses with the solid intraepithelial structure.^[6]^ Third, the “Phrygian cap theory” postulates that during the solid stage, the gallbladder grows at a faster pace than the structure surrounding it.^[7]^ Wrinkling and kinking therefore take place due to lack of space. The “wrinkling theory” and the “Phrygian cap theory” can be deduced by the presence of muscle fibers within the septa.^[6]^

### 3.3 Clinical presentation

Among the pediatric cases, 12 of the 19 cases had biliary symptoms. In the adult population, approximately 71% (n = 27/38 cases) of patients reported biliary symptoms (Table 2A). Regardless of age, among the cases that have detailed descriptions of biliary symptoms (n = 33), upper right quadrant pain was the most common symptom (18 patients [54.5%]), followed by epigastric pain (11 [33.3%]) and nausea/vomiting (9 [27.2%]) (Table 2B). Three pediatric cases had jaundice as one of their clinical presentations, while none of the adults presented with jaundice at diagnosis. An anomalous pancreaticobiliary ductal union, which relates to choledochal cyst and biliary tract carcinoma, was found in a 46-year-old woman with gastric carcinoma, who further showed no tumor involvement in MSG.^[8]^ Three adult cases had a hypoplastic gallbladder, and 4 cases were complicated with gallstones. Additionally, 7 of the 57 patients had cholelithiasis. Three of these cases were found in the pediatric population (Table 1).^[9–11]^

The mechanism of pain is not well understood, but the consensus is that slow bile flow and increased intraluminal pressure lead to the sensation of pain. This might be supported by the delayed passage of bile observed under biliary manometry and scintigraphy.^[12]^ Normally, MSG is not accompanied by malignancy. However, MSG can be complicated by a choledochal cyst or anomalous arrangement of the pancreaticobiliary duct, thereby increasing the risk of malignant transformation.^[13,14]^ Therefore, an advanced evaluation of the associated ductal anomalies should be done. MSG can coexist with choledochal cysts in both pediatric (3/19 cases) and adult (2/38 cases) populations (Table 1).^[15–18]^

### 3.4 Diagnostic approaches

All reported cases were diagnosed using USG. Under USG, with fine echogenic bands arising from the wall and in the absence of acoustic shadowing, the gallbladder would appear to have multiple intercommunicating compartments. Differential diagnosis includes desquamated gallbladder mucosa, polypoid cholesterolosis, hydatid cyst, congenital or acquired intramural diverticulosis, and even acute hepatitis.^[19–23]^

In a 49 years old woman, endoscopic ultrasound was used to confirm the diagnosis of MSG.^[4]^ Singh et al noticed that in cases with partial multiseptate gallbladder, those restricted to the neck of the gallbladder can be fully visualized through endoscopic ultrasound.^[8]^

Oral cholecystography (OCC) was a procedure used to image the gallbladder, which is now largely replaced by ultrasound and MRCP. OCC can show the structure of the gallbladder and the process of gallbladder emptying. In the cases where OCC was used as the imaging tool, the authors reported normal gallbladder contraction.^[24]^ Hepatobiliary iminodiacetic acid scan and biliary manometry with scintigraphy were used to show the bile-excreting function of the liver as well. Results of the hepatobiliary iminodiacetic acid scan showed normal gallbladder emptying, while impairment of gallbladder filling and contraction was revealed on biliary manometry with scintigraphy.^[3,7,12]^

Endoscopic retrograde cholangiopancreatography (ERCP) and MRCP can be used to fully visualize the intra- and extra-biliary tracts. However, ERCP cannot fully establish the MSG structure in some cases.^[14,16]^ In contrast to ERCP, Nakazawa et al suggested that MRCP seems to be a superior and more commonly used imaging modality in recent years due to its non-invasive nature, low radiation, and ability to identify the biliary and pancreatic pathology simultaneously, which affects our treatment decision making.^[14]^ However, adjustments should be made according to hospital resources and weighing the advantages and disadvantages of the patient.

### 3.5 Treatment and prognosis

Excluding 4 cases whose treatment was not described in the articles, about half of the pediatric cases received surgical treatment. Among the 8 children undergoing cholecystectomy, most had biliary symptoms (n = 7/8).

Excision of the extrahepatic biliary tree combined with hepaticojejunostomy, choledochoduodenostomy, or Roux-en-Y anastomosis due to choledochal cyst was done in 3 cases.^[15–17]^ In the 3 patients who had biliary symptoms but chose not to undergo surgical treatment, the symptoms were self-limiting over time.^[10,23,25]^

In adult patients with biliary symptoms, 90% of the adult population underwent surgery. Among them, a 53-year-old woman underwent an additional Roux-en-Y procedure due to co-existing choledochal cysts.

In the case we presented, a 14-year-old girl who had biliary symptoms and was diagnosed with MSG along with gastritis underwent cholecystectomy, and her symptoms persisted after the surgery. This suggests that in the presence of other gastrointestinal conditions, the patient should be treated for such symptoms first while MSG can be managed through active monitoring. Cholecystectomy can be considered after other symptoms are resolved or under control.

## 4 Conclusion

In summary, MSG is a rare congenital biliary anomaly that can occur in children and adults. Most cases are presented with biliary symptoms, but some cases can be asymptomatic. For all MSG cases, it is important to rule out the associated biliary tract anomalies, especially those with a higher risk of malignant transformation. Lab imaging is a vital tool to diagnose MSG and to identify associated biliary tract anomalies. MRCP can be considered a superior imaging modality, such as ERCP, due to its non-invasive property and high resolution of biliary anatomy.

Based on the 57 cases reviewed, asymptomatic cases can remain asymptomatic, and cases with biliary symptoms can recover without treatment. Therefore, regular follow-up is sufficient for asymptomatic MSG without associated biliary tract anomalies. When symptoms occur, they can either be treated with cholecystectomy or left untreated with regular follow-up.

## Acknowledgments

This case report was prepared according to the CARE guidelines.

## Author contributions

**Conceptualization:** Shu-Chao Weng.

**Data curation:** Yu Min Hsieh.

**Supervision:** Nien-Lu Wang, Pao-Shu Wu, Shu-Chao Weng.

**Writing – original draft:** Yu Min Hsieh.

**Writing – review & editing:** Yu Min Hsieh, Yuli Lily Hsieh.
